# Efficacy of apatinib combined with FOLFIRI in the first-line treatment of patients with metastatic colorectal cancer

**DOI:** 10.1007/s10637-021-01205-3

**Published:** 2022-01-08

**Authors:** Xuetong Rong, Haiyi Liu, Hongmei Yu, Jian Zhao, Jie Wang, Yusheng Wang

**Affiliations:** 1 Shanxi Medical University, Taiyuan, Shanxi 030001 People’s Republic of China; 2 Department of Digestive, Affiliated Cancer Hospital of Shanxi Medical University, Taiyuan, Shanxi 030013 People’s Republic of China

**Keywords:** Apatinib, FOLFIRI, Colorectal adenocarcinoma, Survival

## Abstract

*Objective*. To evaluate the efficacy and safety of apatinib combined with FOLFIRI in the first-line treatment of advanced metastatic colorectal cancer (mCRC) and explore potential factors of efficacy. *Methods****.*** Twenty mCRC patients treated at Affiliated Cancer Hospital of Shanxi Medical University from March 2017 to March 2019 were included according to the enrolment criteria. They provided informed consent and were treated with apatinib combined with FOLFIRI according to the scheduled regimen until disease progression or unacceptable toxicity occurred. The primary endpoint was OS. The secondary endpoints included PFS, ORR, DCRand safety. OS and PFS were calculated using Kaplan–Meier curves. Univariate and multivariate Cox regression analyses were used to evaluate independent prognostic factors of OS and PFS. R was used to determine cut-off values for biochemical indicators. Forest maps were drawn for Cox univariate results and the relationships between NLR and ECOG, which were significant in univariate analysis, and OS were represented by Kaplan–Meier curves. *Results****.*** The median OS and PFS were 16.135 months (95% CI: 9.211–22.929) and 6 months (95% CI: 5.425–6.525). Multivariate Cox analysis showed that NLR and CEA were independent prognostic factors. The most common grade 3–4 adverse events were hypertension, diarrhoea, increased alkaline phosphatase, decreased leukocytes and decreased neutrophils. *Conclusion*. Apatinib combined with FOLFIRI for the first-line treatment of advanced unresectable mCRC showed good efficacy and safety. The baseline NLR was predictive of efficacy, and a low baseline NLR (HR: 0.2895, P = 0.0084) was associated with improved OS.

Clinical Research Registration Number: ChiCTR1800015308.

## Introduction

The incidence and mortality of colorectal cancer (CRC) rank third among all malignant tumours worldwide [1]. In China, CRC is the fifth most common malignant tumour [2]. The early diagnosis rate of CRC in China is low. CRC is often in the late stage when diagnosed, with no chance of surgical resection and a short survival period, which seriously threatens the health of people. The treatment of advanced unresectable CRC is mainly systemic treatment. In recent decades, the progress of chemotherapy has promoted the survival of patients with advanced metastatic CRC. Oxaliplatin combined with the 5-FU/ Calcium Folinate regimen (FOLFOX) and irinotecan combined with the 5-FU/ Calcium Folinate regimen (FOLFIRI) are the basic regimens for advanced CRC [3]. The V308 study [4] mentioned that FOLFIRI was approved as the first-line treatment for advanced metastatic CRC by the European and US Food and Drug Administration (FDA) in 2012 [5], and FOLFIRI was also the first recommendation in the Chinese Society of Clinical Oncology (CSCO) guidelines. However, the median survival time of systemic chemotherapy alone was less than 27 months [6]. The advent of targeted drugs has pushed the treatment of advanced CRC to a new stage and extended the survival time of patients. Anti-VEGF and anti-EGFR monoclonal antibodies are currently recognized as targeted therapeutic options for advanced CRC [7], but the anti-EGFR monoclonal antibodies panitumumab and cetuximab have significant effects on patients with wild-type RAS and BRAF, while they have poor effects on patients with mutant RAS and BRAF. Although bevacizumab, an anti-VEGF monoclonal antibody, is effective in the overall population of CRC patients, the toxicities of bleeding, perforation and obstruction cannot be ignored, especially in patients with ulcerative primary lesions, who tend to have severe adverse reactions. Therefore, it is necessary to explore new therapeutic modes to improve the survival of patients with advanced CRC. Apatinib tablet is a small-molecule targeted drug against VEGFR-2 that blocks downstream signal transduction and inhibits tyrosine kinase production through highly selective competition for ATP binding sites of intracellular receptor-2, thus inhibiting neoangiogenesis in tumour tissues and finally achieving the purpose of tumour treatment [8]. Apatinib has a therapeutic effect on various types of cancers. Current exploratory studies of apatinib in advanced CRC focus on later-line treatments. The efficacy of apatinib in first-line treatment is not yet clear. The objective of this open exploratory clinical trial was to evaluate the efficacy and safety of apatinib in combination with FOLFIRI in patients with advanced unresectable metastatic CRC and to explore clinical factors associated with prognosis.

## Materials and methods

1. **Ethics**
  This is a single-centre, single-arm open exploratory clinical trial that has been registered in the China Clinical Research Network (ChiCTR1800015308) and approved by the Ethics Committee of Shanxi Cancer Hospital under careful review (Approval number: 201719).
2. **Patients and grouping**
  From March 2017 to March 2019, all patients received treatment at the Affiliated Cancer Hospital of Shanxi Medical University. The inclusion criteria were as follows. Patients were aged 18–75 years, and there were no limitations on sex. Patients were diagnosed with colorectal adenocarcinoma with concurrent distant metastasis by pathology or patients had undergone radical resection of their primary tumour. According to Response Evaluation Criteria in Solid Tumors (RECIST) 1.1, patients had at least one measurable target lesion (tumour lesion 10 mm long diameter on computed tomography (CT) scan, lymph node lesion 15 mm short diameter on CT scan, scan thickness no greater than 5 mm, and no local treatment). Patients had an expected survival time of 3 months. Patients had an Eastern Cooperative Oncology Group (ECOG) physical condition score of 0–2. The function of major organs was good; that is, the relevant examination indexes within 14 days before enrolment met the following requirements: A. routine blood examination: haemoglobin 90 g/L; neutrophil count 1.5 10^9/L; platelet count 100 10^9/L; and white blood cell count 3.5 10^9/L; B. biochemical examination: total bilirubin 1.5 upper limit of normal (ULN); alanine aminotransferase (ALT) or aspartate aminotransferase (AST) 2.5 ULN; ALT or AST 5 ULN if liver metastases;serum creatinine (Cr) 1 ULN, endogenous Cr clearance rate 50 mL/mins (Cockcroft-Gault formula); women of childbearing age must have a negative pregnancy test (serum or urine) within 7 days prior to enrolment and voluntarily use an appropriate method of contraception during observation and 8 weeks after the last administration of apatinib mesylate tablets. The subjects voluntarily joined the study and signed the informed consent form.
3. **Treatment regimen**
  All subjects received the first-line treatment regimen of apatinib combined with FOLFIRI: apatinib tablet 250 mg once a day, orally with warm water half an hour after a meal (the time the drug is taken should be the same as much as possible). After one week, if the adverse reactions were well tolerated, the dose was adjusted to 500 mg once a day orally. In the case of grade III adverse reactions, the dose could be reduced to 250 mg once a day. Treatment discontinuities due to adverse events were permitted for no more than 14 days. Subjects were treated until disease progression or death from unacceptable toxicity, and the apatinib FOLFIRI regimen was as follows: irinotecan 180 mg/m^2^ continued intravenously for 90 min, day 1; calcium tetrahydrofolate 400 mg/m^2^, intravenous infusion (2 h), day 1; fluorouracil 0.4 g/m^2^ intravenously (1 h after calcium tetrahydrofolate); fluorouracil 2.4 g/m^2^, continuous intravenous infusion (chemotherapy pump infusion) for 46 h, repeated every 14 days.
4. **Data collection**
  Before treatment, routine blood biochemical indicators, such as coagulation function indicators, were collected, and the neutrophil-to-lymphocyte ratio (NLR) in routine blood was calculated as the absolute count of neutrophils divided by the absolute count of lymphocytes. All subjects underwent CT 1 cycle after baseline, every 2 cycles thereafter and at disease progression. Overall survival (OS) was defined as the time from randomization to death from any cause. Progression-free survival (PFS) was defined as the time from randomization to tumour progression or death. Subjects must have a measurable tumour focus at baseline. Efficacy was assessed as complete response (CR), partial response (PR), stable disease (SD), and progressive disease (PD) according to RECIST 1.1. The objective response rate (ORR) is the proportion of patients whose tumours shrank to a certain extent and remained so for a certain period of time, including those with CR and PR. The disease control rate (DCR) was defined as the proportion of patients with CR, PR, and SD. Adverse events were graded from 0 to 4 based on the National Cancer Institute Common Terminology Criteria for Adverse Events (NCI-CTCAE 4.0).
5. **Statistical analysis**
  The baseline demographics and disease characteristics were treated as categorical variables. R 3.4.4 software was used to determine thresholds for the baseline NLR, carbohydrate antigen 19–9 (CA19-9) and carcinoembryonic antigen (CEA) levels through the survivalROC package. The Kaplan–Meier (KM) method was used to analyse OS and PFS. A Cox proportional hazards regression model was used for univariate and multivariate analyses, the univariate analysis results were displayed in a forest map, and the variables with statistical significance in the univariate analysis were plotted with KM curves. The results included curves of the relationship between NLR and OS and the relationship between ECOG and OS. A variable with P value < 0.20 in univariate analysis was set as a covariable, and P-value < 0.05 was considered statistically significant in the multivariate analysis. Finally, the statistically significant results are presented in a table. All statistical estimates were two-sided, and P-value < 0.05 was considered statistically significant. SPSS version 26.0 software and RStudio were used for the analyses, and the R packages used included forestplot, grid, magrittr, and checkmate.

## Results

**Fig. 1:**
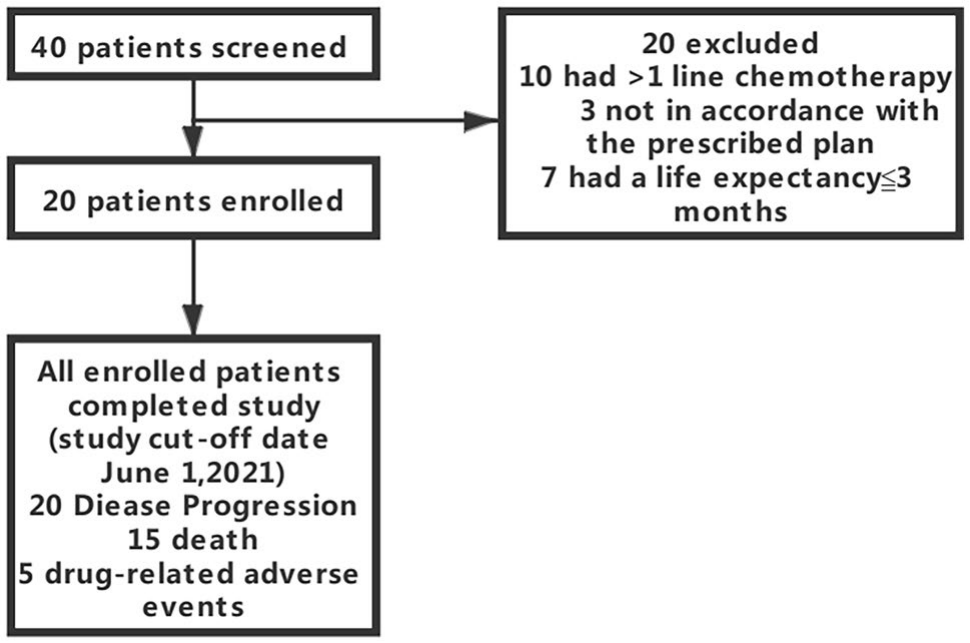
CONSORT diagram of study population selection for chemotherapy-refractory metastatic colorectal cancer

1. **Patient characteristics**
  From March 2017 to March 2019, 40 patients were screened, and a total of 20 subjects met the inclusion criteria (Fig. 1). As of June 1, 2021, the median follow-up time was 21.9 months (7.73 months-36.23 months). Among the 20 registered subjects, 20 (100%) withdrew from the study due to disease progression, of which 15 died (75%) and 5 (25%) had drug-related adverse events. The baseline demographics and pretreatment characteristics are shown in Table 1. The median age was 59.5 years (44–71 years), and approximately half of the subjects were male (12[60%]). About one-third of the subjects had an ECOG performance status score of 2. Most subjects had multiple sites of metastasis (15 [75%]), and the liver and lung were the most common sites of metastasis (20 [100%]). None of the subjects had received any antitumour treatment before enrolment.

**Fig. 2:**
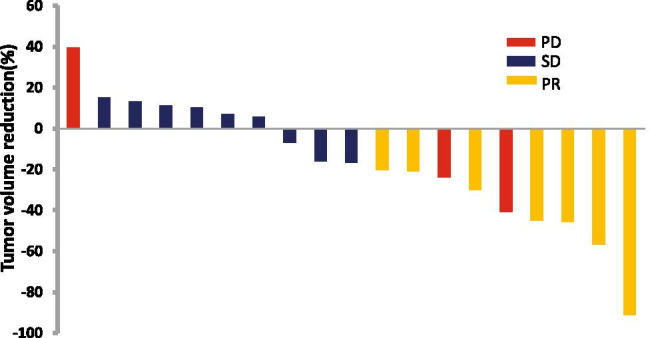
Waterfall plot of best percentage change from baseline in measurable tumour lesions

**Fig. 3:**
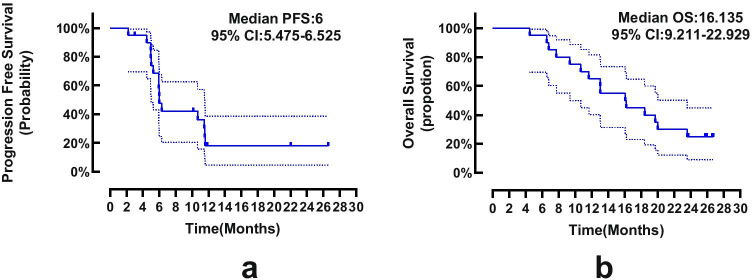
Kaplan–Meier curves for PFS (**a**) and OS(**b**) of the enrolled patients. PFS, progression-free survival; OS, overall survival; CI, confidence interval

**Fig. 4:**
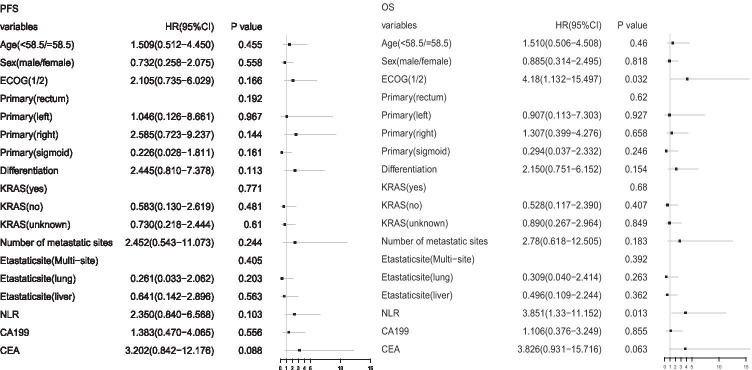
Univariate analysis of factors to predict progression-free survival and overall survival of apatinib shown by forest plot. PFS, progression-free survival; OS, overall survival; ECOG, Eastern Cooperative Oncology Group; NLR, neutrophil-to-lymphocyte ratio; CEA, carcinoembryonic antigen; CA19–9, carbohydrate antigen 19–9; HR, hazard ratio; CI, confidence interval; KRAS(yes), KRAS mutation; KRAS(no), no KRAS mutation; KRAS(unknown), unknown KRAS mutation status

**Table 1:** Patients characteristics

Patient characteristic	Value (n. %)
Median age, years (range)	59.5(44–71)
Sex	
Male	12(60)
Female	8(40)
ECOG performance status	
1	14(70)
2	6(30)
Primary tumour location	
Left	1(5)
Right	5(25)
Rectum	11(55)
Sigmoid colon	3(15)
Differentiation	
Moderate	14(70)
Low	6(30)
KRAS mutation	
No	5(25)
Yes	5(25)
Unknown	10(50)
Metastatic site	
Lung	11(55)
Liver	15(75)
Multi-site	14(70)
NLR	
< 3.855	12(60)
≥ 3.855	8(40)
CA199	
< 428.735	17(85)
≥ 428.735	3(15)
CEA	
< 39.645	13(65)
≥ 39.645	7(35)
Surgery	
Radical mastectomy	6(30)
Palliative surgery	8(40)
None	6(30)

Table.1 Patients’ baseline characteristics (N = 20)

*ECOG* Eastern Cooperative Oncology Group, *NLR* neutrophil-to-lymphocyte ratio, *LDH* lactate dehydrogenase, *CEA* carcinoembryonic antigen, *CA19–9* carbohydrate antigen 19–9

2. **Efficacy analysis**
  Seven subjects (35%) achieved PR, nine subjects (45%) achieved SD, and three subjects (15%) developed PD. The results of the efficacy analysis are shown in Fig. 2. One subject did not undergo CT examination during the second treatment cycle, so an efficacy evaluation could not be performed.

3. **Survival analysis**
  At the data cut-off, all enrolled subjects had completed treatment and were available for survival analysis. The median PFS and OS were 6 (95% confidence interval (CI) 5.475–6.525) and 16.135 (95% CI 9.211–22.929) months, respectively (Fig. 3).

4. **Safety analysis**
  The overall adverse event rate was 100% in the 20 subjects, but the majority of patients experienced grade 1 or 2 toxicities; 50% of the patients experienced hypertension, nausea, fatigue, hand-foot skin reaction, or leukopenia; and 6 (30%) subjects had grade 3–4 adverse events, including hypertension, diarrhoea, alkaline phosphatase increase, leukopenia, and neutropenia. The adverse events are shown in Table 2.

**Table 2:** Adverse events

Adverse event	Grade 1–2 (n,%)	Grade 3–4 (n,%)	Total(n,%)
Nonhaematologic			
Hypertension	16(80)	2(10)	18(90)
Vomiting	18(90)	0	18(90)
Fatigue	13(65)	0	13(65)
Hand-foot syndrome	13(65)	0	13(65)
Abdominal pain	4(20)	0	4(20)
Diarrhoea	2(10)	1(5)	3(15)
Bleeding	4(20)	0	4(20)
Mucositis oral	6(30)	0	6(30)
Proteinuria	7(35)	0	7(35)
Serum AST increased	5(25)	0	5(25)
ALP increased	1(5)	1(5)	2(10)
Hyperbilirubinemia	9(45)	0	9(45)
Haematologic			
Leukopenia	15(75)	1(5)	16(80)
Neutropenia	8(40)	1(5)	9(45)
Thrombocytopenia	7(35)	0	7(35)

Table.2 Adverse events in the whole cohort (n =20)

**Fig. 5:**
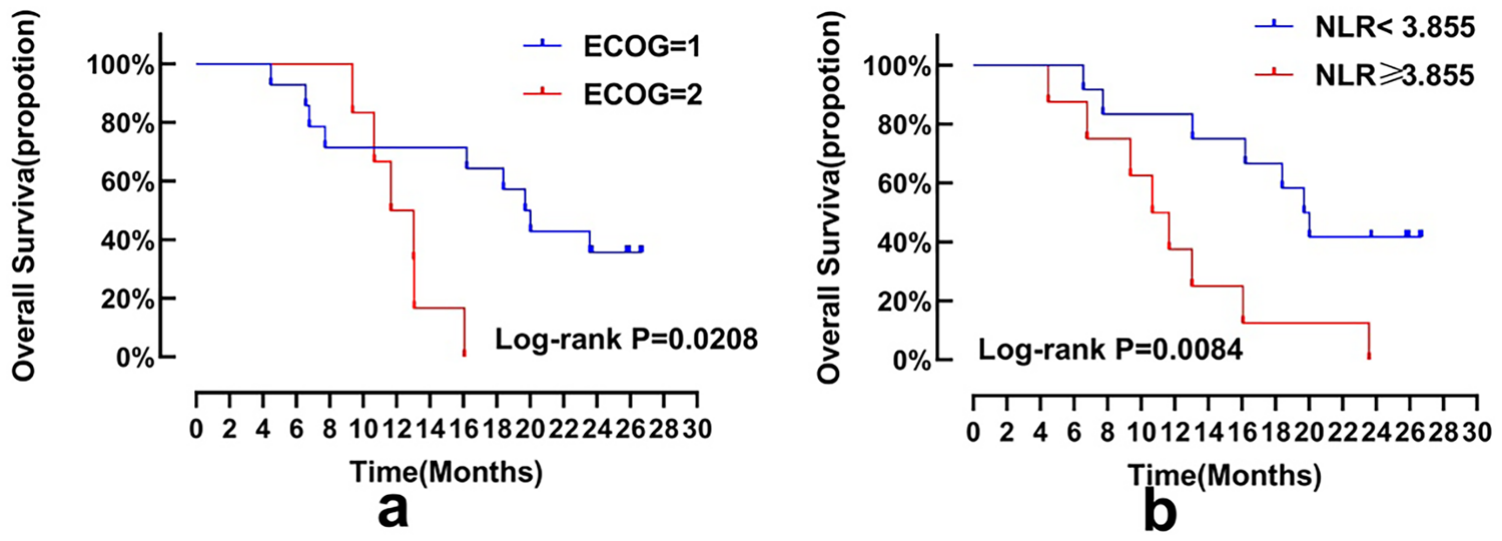
Kaplan–Meier estimates of subgroup analyses. Notes: (**a**) OS and (**b**) OS were estimated for patients with different ECOG and NLR levels. NLR, neutrophil-to-lymphocyte ratio; ECOG, Eastern Cooperative Oncology Group; OS, overall survival

5. **Exploratory analysis of molecular markers**
  The relationship between clinical outcomes and several variables (including baseline characteristic laboratory parameters and drug-related adverse events) was analysed in the enrolled subjects. Receiver operating characteristic (ROC) curves were used to determine critical values. The threshold for NLR was 3.855 to distinguish between low (NLR & LT 3.855) and high (NLR 3.855) subjects, the threshold for CA19-9 was 428.735 to distinguish between low (NLR & LT 428.735) and high (NLR 428.735) subjects, and the threshold for CEA was 39.645 to distinguish between low (NLR & LT 39.645) and high (39.645) subjects. The area under the curve (AUC) for NLR was 0.7867, the AUC for CA19-9 was 0.6, and the AUC for CEA was 0.5467. Subject characteristics, including age, sex, tumour location, degree of differentiation, KRAS gene mutation status, number of metastatic sites, metastatic sites and CA19-9, were independent predictors of PFS and OS (Fig. 4). The relationship between ECOG and OS (P = 0.032) and the relationship between NLR and OS (P = 0.013), which were significant in the univariate analysis, were visualized by the KM method (Fig. 5). In the multivariate Cox regression analysis, low baseline NLR (hazard ratio (HR): 9.767, 95% CI: 1.333–71.554, P = 0.025) and low baseline CEA (HR: 17.691, 95% CI: 1.163–269.081, P = 0.039) were significantly correlated with increased OS, as shown in Table 3. The results of multivariate analysis showed that NLR and CEA are independent prognostic factors of OS.

**Table 3:** Multivariate analysis

Variable	PFS		OS	
	HR (95%CI)	P value	HR (95%CI)	P value
NLR				
< 3.85/≧3.855	3.115(0.533–18.195)	0.207	9.767(1.333–71.554)	0.025
CEA				
< 39.645/≧39.645	5.824(0.518–65.419)	0.153	17.691(1.163–269.081)	0.039

Table.3 Multivariate analysis of factors to predict progression-free survival and overall survival of apatinib

*PFS* progression-free survival, *OS* overall survival, *NLR* neutrophil-to-lymphocyte ratio, *CEA* carcinoembryonic antigen, *HR* hazard ratio

## Discussion

The incidence of CRC ranks third among all malignant tumours, and it is the fourth most deadly cancer worldwide [9]. The incidence and mortality of CRC in Asia have shown upward trends [10]. The early diagnosis rate of CRC in mainland China is low, and the disease is often in the late stage when diagnosed. At that point, the opportunity for surgical resection has been lost, the survival rate is low, and the mortality rate is high. Targeted therapy combined with chemotherapy has significantly improved the survival of patients with advanced CRC [11]. The targeted drugs for CRC that have been approved in China are bevacizumab and cetuximab. Cetuximab is mainly used for KRAS, NRAS, and BRAF wild-type CRC. Bevacizumab has obvious adverse reactions when applied to patients with bleeding, obstruction, and perforation tendencies, so many factors should be considered in the clinic when deciding which targeted drugs should be used. FOLFOX and FOLFIRI are commonly used options for metastatic CRC, and the two are used equivalently in first-line treatment. The two chemotherapy regimens have their own adverse reaction spectrum and are applied to different groups of people. Targeted drugs combined with chemotherapy regimens are currently the main means for treating advanced CRC. The selection of suitable targeted drugs and chemotherapy regimens is the main factor affecting the survival of advanced CRC patients. There are still many patients in the clinic who cannot be treated with the above two targeted drugs due to various factors, so more treatment methods need to be explored.

Apatinib is a small molecule inhibitor that specifically binds to vascular endothelial growth factor receptor 2 (VEGFR-2). Blocking VEGFR-2 can inhibit the migration and proliferation of vascular endothelial cells and reduce the microvessel density of tumours, thereby inhibiting tumour angiogenesis and exerting antitumour effects [12, 13]. Apatinib can enhance endoplasmic reticulum autophagy by enhancing the stress of oestrogen receptors to promote proteasome degradation, thereby inducing the apoptosis of CRC cells [14]. At the same time, apatinib inhibits the AKT-mTOR signalling pathway and increases the expression of Lc3-II to induce autophagy. In the process of autophagy, the UIK1 autophagy complex can be positively regulated by inhibiting mTORC1, thereby promoting autophagy and apoptosis [15]. Studies have shown that apatinib increases the ubiquitination of β-catenin and reduces the phosphorylation of Ser-9 in GSK3β, thereby reducing the nuclear localization of β-catenin and inhibiting the VEGFR2-β-catenin pathway to exert an antitumour effect [16]. Studies have shown that apatinib monotherapy for the late-line treatment of patients with advanced metastatic CRC who have failed chemotherapy has shown a good clinical effect compared with the control. The median PFS was 2 months, and the OS was 5 months longer than that of the control observation group. It is believed that apatinib has a certain clinical effect on the improvement of OS [17]. Therefore, we designed the regimen of apatinib combined with FOLFIRI to explore the efficacy and safety of this regimen as first-line therapy for advanced metastatic CRC.

In our study, the median OS of apatinib combined with FOLFIRI in the treatment of advanced unresectable CRC was 16.135 months (95% CI: 9.211–22.929), and the median PFS was 6 months (95% CI: 5.475–6.525). The ORR was 35%, and the DCR was 80%. The common adverse events were hypertension (90%), vomiting (90%), fatigue (65%), hand-foot syndrome (65%), white blood cell reduction (80%), neutropenia (45%), and hyperbilirubinemia (45%). Serious adverse events (grade > 3) were hypertension (11.1%), diarrhoea (5%), increased ALP (5%), leukopenia (5%), and neutropenia (5%). This study shows that the combination of apatinib and FOLFIRI as a first-line treatment for advanced unresectable metastatic CRC has good efficacy and tolerable adverse reactions. Compared with the previous treatment data of regorafenib, the first-line application of an apatinib combination regimen can give patients a better benefit. In the global CORRECT [18] study, regorafenib was used as a third-line targeted drug to treat advanced metastatic CRC in subjects who were frail or unsuitable for treatment. The median PFS was 1.9 months, and the OS was 6.4 months, which were improved compared to those of previous placebo treatment and best supportive treatment. In the CONCUR study, the median PFS and OS of Asians using regorafenib were 3.2 months and 8.8 months, respectively [19]. In clinical studies of regorafenib as a first-line application, the median PFS was 5.6 months, and the median OS was 16 months [20]. Our results are better than the first-line treatment data of single-agent regorafenib. The subjects we enrolled had the following characteristics: all subjects were stage IV patients at the time of enrolment, 14 (70%) subjects had more than two metastatic sites, and of 15 (75%) subjects with simultaneous liver metastases, 11 (55%) subjects had lung metastasis, and 6 (30%) subjects had an ECOG performance status score of 2 points. The liver is the most common site of metastasis in patients with advanced CRC. Approximately half of CRC patients will develop liver metastasis because most of the mesentery drains into the hepatic portal vein system [21], and liver invasion and metastasis can lead to poor prognosis [22]. The lobe of the lung is the second most common metastatic site of CRC after the liver [23], and lung metastasis develops more slowly than liver metastasis. This study included many patients with multisite metastases. At the time of enrolment, the subjects had a poor physical condition and poor tolerance, so the prognosis was poor. However, after the administration of apatinib combined with the FOLFIRI regimen, a certain curative effect was achieved, and there were tolerable adverse reactions.

In our study, the subjects’ OS varied in length, with the longest being 26.7 months and the shortest being 4.47 months. For this reason, an effective predictive marker must be identified. There has been much research evidence that the NLR is significantly related to the prognosis of CRC. The NLR is a potential prognostic and predictive factor for a variety of tumours, including CRC [24]. In the tumour microenvironment, genes encoding tumour-related macrophage activities, such as the LYZ (lysozyme), TYMP (thymidine phosphorylase), and CD68 (pan-macrophage) genes, may affect the NLR. TYMP promotes angiogenesis, escapes apoptosis, and stimulates tumour growth [25], and the accumulation of high levels of macrophages is positively correlated with high NLR levels. Neutrophils secrete a variety of proinflammatory, angiogenic and immunomodulatory factors, which have paracrine effects on tumour cell biology [26]. Lymphocytes induce cytotoxic cell death and produce cytokines that inhibit the proliferation and metastasis of cancer cells, thereby playing an important role in tumour suppression [27]. Neutrophils can inhibit the cytolytic activity of lymphocytes to tumour cells. In CRC, an increase in the NLR value has an adverse effect on the survival rate of patients with CRC. However, this molecular mechanism is still complex and uncertain [28]. High NLR is associated with a highly aggressive tumour phenotype, and the degree of systemic inflammation is related to the subject's own physical state, which may weaken the subject's own tolerance and compliance, leading to reduced survival rates [29]. Our results show that the prognosis of subjects with NLR < 3.855 is better than that of subjects with NLR ≥ 3.855. Therefore, the NLR at baseline is an independent predictor of the OS of subjects treated with apatinib.

This study is a rigorously executed prospective study with strict ethical review. However, because one-third of the subjects in our clinical study had a baseline ECOG performance status score of 2 with simultaneous multisite metastases, the physical status of patients was relatively poor. Thus, the factors related to the poor prognosis of the subjects and biased enrolment led to unsatisfactory endpoints of OS and PFS in the study. In particular, the mismatch repair protein, RAS, RAF, and HER2 statuses were not standardized during enrolment. Accurate diagnosis has certain limitations. Our study is a single-arm design, and the small sample size affects the objectivity of our data. In the future, we will continue to carry out prospective randomized controlled clinical studies to include more subjects in strict accordance with the inclusion criteria, verify the efficacy and safety of apatinib combined with the FOLFIRI regimen, and further explore the relationship between the NLR and the curative effect of this treatment regimen.

## Conclusions

This is a prospective, single-arm, single-centre, open phase II clinical study confirming that apatinib combined with the FOLFIRI regimen has a better curative effect and tolerance in patients with poor physical fitness and advanced unresectable CRC. It has an acceptable safety profile. It was verified that the baseline NLR can predict the efficacy of apatinib tablets combined with the FOLFIRI regimen. Exploratory research was performed to further identify treatment options for advanced unresectable CRC.

## Data Availability

The data that support the findings of this study are available from the corresponding authors upon reasonable request.
